# Supplementary material for: What Difference Does it Make? Risk-Taking Behavior in Obesity after a Loss is Associated with Decreased Ventromedial Prefrontal Cortex Activity
Source: J Clin Med. 2019 Sep 27;8(10):1551. doi: 10.3390/jcm8101551 (PMC6832276; doi:10.3390/jcm8101551)
Supplement: Supplementary file 1 [file jcm-08-01551-s001.zip › jcm-578485-SI.pdf]

Supplementary Information

Inclusion and exclusion criteria

The inclusion criteria for this study were the following: being female and being between the age of 18 and 55. Healthy control (HC) participants were required to have a body mass index (BMI) between 18.5 and 25.0 and participants with obesity (OB) were required to have a BMI over 30. The study exclusion criteria were being male, the presence of an organic mental disorder or an intellectual disability, a current eating disorder or other psychiatric disorder (psychotic disorders, bipolar disorder, substance dependence, or anxiety and depressive disorders). Women who were pregnant or currently breastfeeding were not allowed to participate in the study. Prior to assessment, HC participants were asked to report maximum lifetime BMI and those who endorsed having had obesity (BMI>30) were excluded from the study sample. HC participants received compensation (€80) for participating in the study.

Figure S1. Shared task activation patters in the obesity and control groups during loss vs. win trials of the Risky Gains Task.

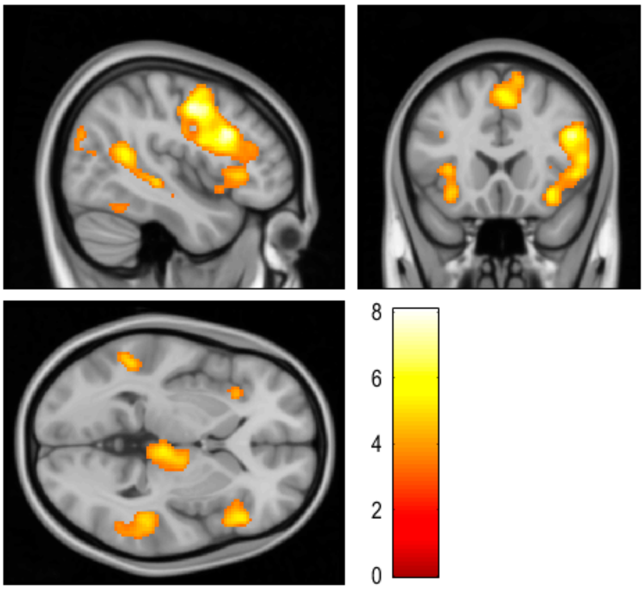

Increased activation was found in the precuneus, the dorsomedial and dorsolateral prefrontal cortex, and the anterior insula during the losses vs. wins contrast of the Risky Gains Task ($p < 0.001$, uncorrected). Color bar represents t-values.

Figure S2. Shared task activation patters in the obesity and control groups during risky vs. safe trials of the Risky Gains Task.

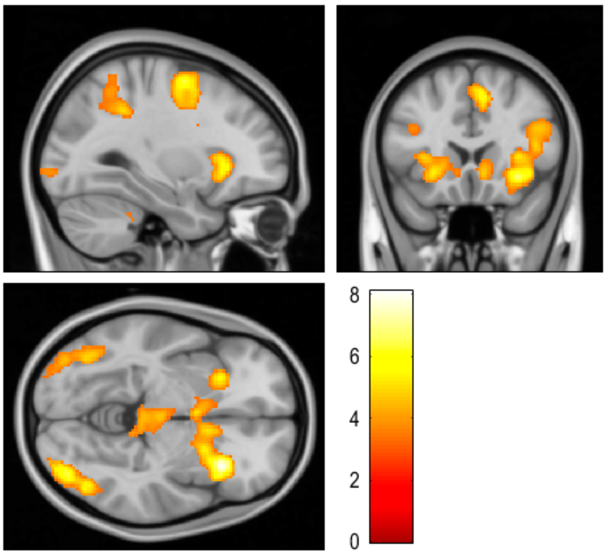

Increased activation was found in the anterior insula, the caudate nucleus and a cluster comprising the bilateral inferior frontal gyrus during the risky vs. safe contrast of the Risky Gains Task ($p < 0.001$, uncorrected). Color bar represents t-values.

Figure S3. Association between insula peak activations and UPPS-P sensation seeking scores in the HC group and whole sample.

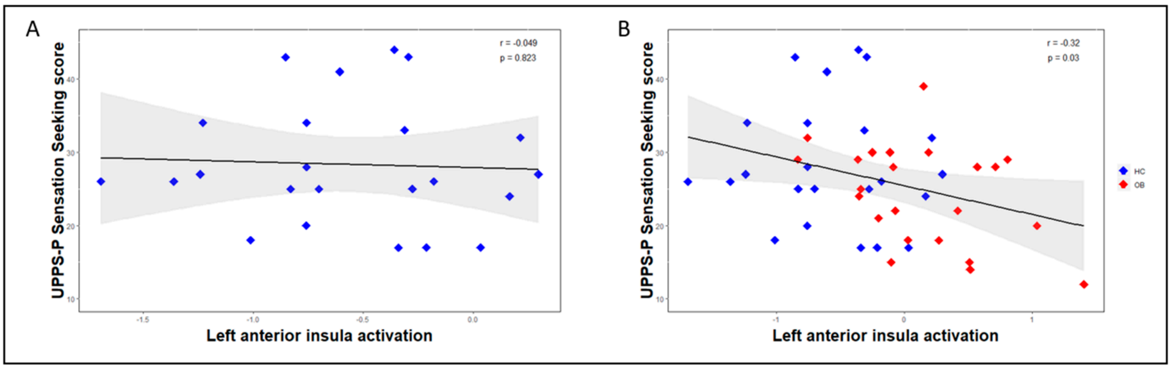

A scatterplot depicting the association between extracted activation eigenvalues from the left anterior insula peak (losses vs. wins) and UPPS-P sensation seeking scores in (A) healthy controls and [n=23, r(23) =-0.049, p=0.823] (B) the whole sample [n=46, r(46) =-0.320, p=0.030].

Figure S4. Association between vmPFC peak activation and the percentage of risky choices in the HC group and whole sample.

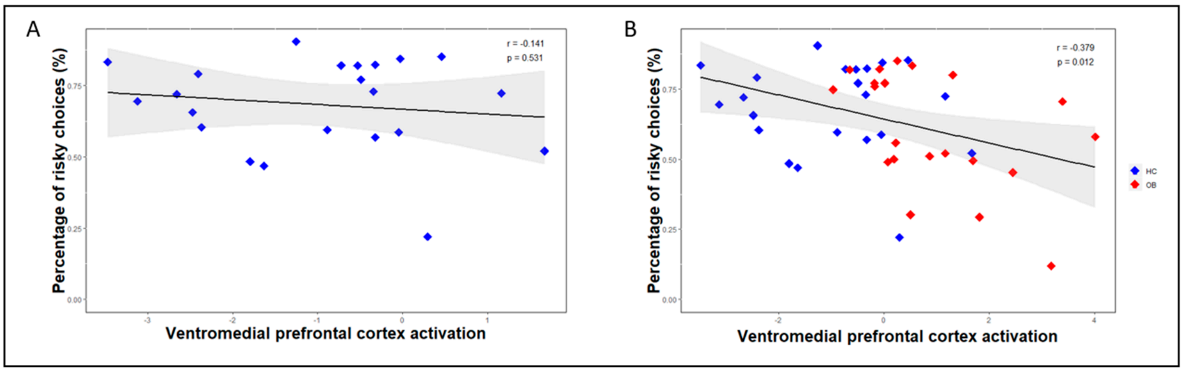

A scatterplot depicting the correlation between extracted activation eigenvalues from the vmPFC peak and percentage of total risky choices in (a) healthy controls [n= 22, (r(22)= -0,141, p=0.531)] and (B) the whole sample [n= 43, (r(43)= -0,379, p=0.012)] .
